# The impact of digital finance on household participation in risky financial markets: Evidence-based study from China

**DOI:** 10.1371/journal.pone.0265606

**Published:** 2022-04-07

**Authors:** Yun Ye, Yongjian Pu, Ailun Xiong

**Affiliations:** 1 School of Economics and Business Administration, Chongqing University, Chongqing, China; 2 School of Management, Chongqing Technology and Business University, Chongqing, China; BeiHang University School of Economics and Management, CHINA

## Abstract

Chinese households have overinvested in risk-free financial assets, resulting in a single structure of financial assets. However, this proportion declined as digital finance developed. By combing the data from the China Household Finance Survey Data and the Peking University Digital Financial Inclusion Index of China, we find that digital finance significantly promotes household participation in risky financial markets. Further mechanistic analysis unveils that digital finance mainly affects households’ participation in risky financial markets by reducing the lack of investment channels, promoting households’ access to financial information and increasing the possibility of household risk appetite. In addition, the heterogeneity analysis suggests that digital finance effectively reduces the deterrent effect of wealth and cognitive thresholds on all households, reflecting the inclusive nature of financial development. Our findings provide an empirical basis for the mainstream positioning of digital finance in the development of inclusive finance, and display its positive impact on social welfare.

## 1 Introduction

China’s household asset allocation structure has long been unitary. This phenomenon manifests as investing most of their assets in risk-free financial assets such as deposits [1]. This asset allocation strategy makes households’ finances inefficient because it incurs investment risks that are too low. Additionally, it is not conducive to smoothing household consumption [2] and reducing income inequality [3, 4]. Diversification is an important way to promote reasonable risk taking by households [5]. However, Chinese households do not fully benefit from the simple principle and are significantly underdiversified. According to China’s Household Financial Survey Report, there has been an increase in household participation in risky financial markets in the past 3 years; however, it is still a tiny number. As of 2019, risk-free assets accounted for >75% of China’s household financial assets. The proportions of households holding stocks, funds and bonds in China were 4.4%, 1.3% and 0.2% respectively. Conversely, data from the US Consumer Finance Survey show that the proportions of U.S. households holding stocks, funds and bonds were 15.2%, 9.0% and 8.6% respectively. Therefore, the financial market participation of Chinese households is insufficient. This lack of participation not only severely hinders the growth of household wealth, but also leads to the loss of general social welfare. In this regard, studying household financial asset allocation behavior can help guide households to rationally participate in venture capital and achieve wealth goals. It is also of great significance for the development of China’s financial market and the formulation of relevant policies for financial innovation.

Existing studies mainly discuss the possibility of household participation in the financial markets from such micro perspectives of household demographic characteristics as [6], social capital [7], housing assets [8] and so on, but seldom consider the impact of the macro environment. However, this paper finds that since the launch of Yu’E Bao in 2013, China’s digital finance has developed rapidly, with the Peking University Digital Financial Inclusion Index (PKU-DFIIC) rising from 155 in 2013 to 228 in 2017. Meanwhile, calculations based on data from the Chinese Household Finance Survey (CHFS) show that the participation rate of Chinese households in risky financial markets rose from 2% in 2013 to 26% in 2017, which is basically in line with the development trend of digital finance. Therefore, can the rapid development of digital finance help Chinese households participate in risky financial markets? Moreover, how will digital finance affect household participation in risky financial markets? To date, limited studies have conducted direct quantitative evaluations based on microdata.

Digital finance, with its outstanding features of low cost, wide- coverage, and high efficiency, has effectively promoted the development of financial products and services [9, 10]. Therefore, digital finance may affect household financial asset allocation in many ways. First, digital finance breaks through the geographical limitations of traditional financial transactions and brings time and space convenience to family investment, especially in remote areas [11]. In addition, the rapid penetration of digital finance has spawned a large number of internet financial products, which have broadened family investment channels and given households more investment opportunities. Second, digital finance uses efficient features of internet technology to share economic information, thus reducing information asymmetry and transaction costs [12]. With this advantage, digital finance promotes households’ access to financial information and helps households make financial asset allocation decisions. Third, with the rapid penetration of digital finance into every aspect of family life, households have various financial opportunities, such as mobile payment and online shopping [13]. In the process of using digital financial products and services, households have raised their risk-bearing level to some extent [14], thereby motivating them to participate in risky financial markets.

From the above theories, we argue that digital finance in China has effectively promoted household participation in risky financial markets by alleviating various transaction barriers. To verify this hypothesis, we combine data from the CHFS and PKU-DFIIC to explore the impact of digital finance on household participation in risky financial markets. Our findings show that digital finance significantly promotes household participation in risky financial markets. This finding still holds after considering the problem of endogeneity and a series of robustness tests. Moreover, the mechanism analysis reveals that the development of digital finance significantly reduces the lack of investment access, promotes households’ access to financial information and increases the possibility of household risk appetite. Further heterogeneity analysis finds that the development of digital finance effectively reduces the deterrent effect of wealth and cognitive thresholds, again reflecting the inclusive characteristic of digital finance. However, the digital divide still exists, which the relevant department should pay attention to.

Considering that the financial environment is changing in the era of the digital economy, our study primarily focuses on the impact of digital finance on household participation in risky financial markets. Therefore, this study may contribute to the existing literature in the following ways. First, it contributes to the literature on digital finance. Although scholars have studied the positive effects of digital finance on household consumption [15], poverty alleviation [16], income growth [17] and risk-taking [13], few studies have discussed the role played by digital finance in households’ participation in risky financial markets. We show that digital finance significantly promotes the probability of household participation in risky financial markets. Second, our study adds to the existing literature by providing a new perspective on household financial behavior. Despite increasing research being conducted on household asset portfolios, most studies are rooted in developed countries, both theoretically and empirically [18]. Previous studies have mainly shown the micro perspectives of household investment behavior, and macro perspectives have received little attention. Thus, we explore the manifestations and factors that influence household financial behavior from the macro perspective, which enriches the current household-finance literature. Finally, we also analyze the group differences and regional differences of digital finance affecting household financial participation and verify the impact mechanism from three aspects: household investment convenience, financial information access and risk-taking level, which broadens the research scope and deepens the policy connotation of this paper.

The remainder of the paper is organized as follows. Section 2 presents a literature review and hypotheses. Section 3 introduces the survey data and models and presents the variable statistics. Section 4 reports the empirical results. Section 5 concludes and discusses policy implications.

## 2 Literature review and hypotheses

### 2.1 Literature review

Many scholars have studied the factors affecting household investment behavior, mainly from the dimensions of household demographic characteristics, background risks, and economic characteristics. First, the existence of household heterogeneity makes households allocate financial assets according to their own characteristics, such as age [19], education [20], household wealth [7], and risk attitude [21]. Second, households often face background risks, such as labor income risk [2], health risk [22], and commercial and their own-residential property investment risks [23, 24]. Background risks are generally considered to be risks that cannot be dispersed through simple financial means such as investment diversification and flexible allocation [25]. Third, market friction is an important factor affecting household investment behavior. It mainly includes transaction costs and information costs to limit participation opportunities [26]. The transaction costs mainly refers to the fixed cost of entering a risky financial market, and is an important reason for the low level of participation of low-income households [27]. In addition, high information search costs hinder households from participating in risky financial markets [28].

Some scholars have found that financial development is also an important factor affecting household investment behavior. Some important reasons for insufficient participation might be the product supply structure of the financial market and investment barriers [29]. As a combination of the internet and finance, digital finance naturally has financial characteristics. Digital finance relies on digital technology to provide better payment services, credit services, financing services, etc., thus achieving high-quality financial services such as reducing information costs [30] and broadening the boundaries of financial services [31]. Accordingly, some studies highlighted the importance of the information function and other features of digital finance [32]. First, digital finance promotes the development of financial inclusion through high-quality development [33], poverty reduction [34] and economic growth [35] while expanding the availability of financial services to vulnerable groups excluded by traditional finance [36]. Second, digital finance supports consumption [37], entrepreneurship [38] and urbanization [39] by increasing connectivity and reducing the digital divide. Third, digital finance plays an important role in promoting the further development of traditional finance [40]. Its high efficiency and low cost advantages not only help traditional finance improve service efficiency [41, 42], but also help optimize management and innovate products [43].

In addition, digital finance plays an increasingly greater role in household investment behavior. On the one hand, digital finance, through its inclusive nature, makes up for the shortage of bank branches in rural areas and provides equal opportunities for rural areas to obtain financial services [44]. On this basis, it improves the possibility of peasant households participating in risky financial markets and the allocation proportion of risky financial assets [45]. Other scholars further analyzed the regulatory mechanism of digital finance on promoting household participation in risky financial markets, mainly including educational cognition and household income. From the perspective of the economic status of men and women, some studies empirically concluded that the inclusion of digital finance helps to improve women’s financial cognition, thus promoting family participation in venture capital [46, 47]. Furthermore, studies from other groups claimed that digital finance has increased the household income of rural residents by supporting entrepreneurship and alleviating liquidity constraints. In particular, the income-increasing impact of digital finance is higher in rural low-income groups, which in turn promotes household participation in risky financial markets [48]. Of note, higher incomes make transaction cost more affordable for households; thus, higher incomes encourage households to allocate more to risky financial assets [49, 50]. However, the relevant research objects are mostly targeted at farmers, who seem to be more able to realize its universal benefits. Indeed, urban households should also be the beneficiaries of digital finance. Therefore, the existing research still has room for expansion. In addition, the influence channels of digital finance are diverse, which is worth further exploration.

### 2.2 Hypotheses

**Fig 1:**
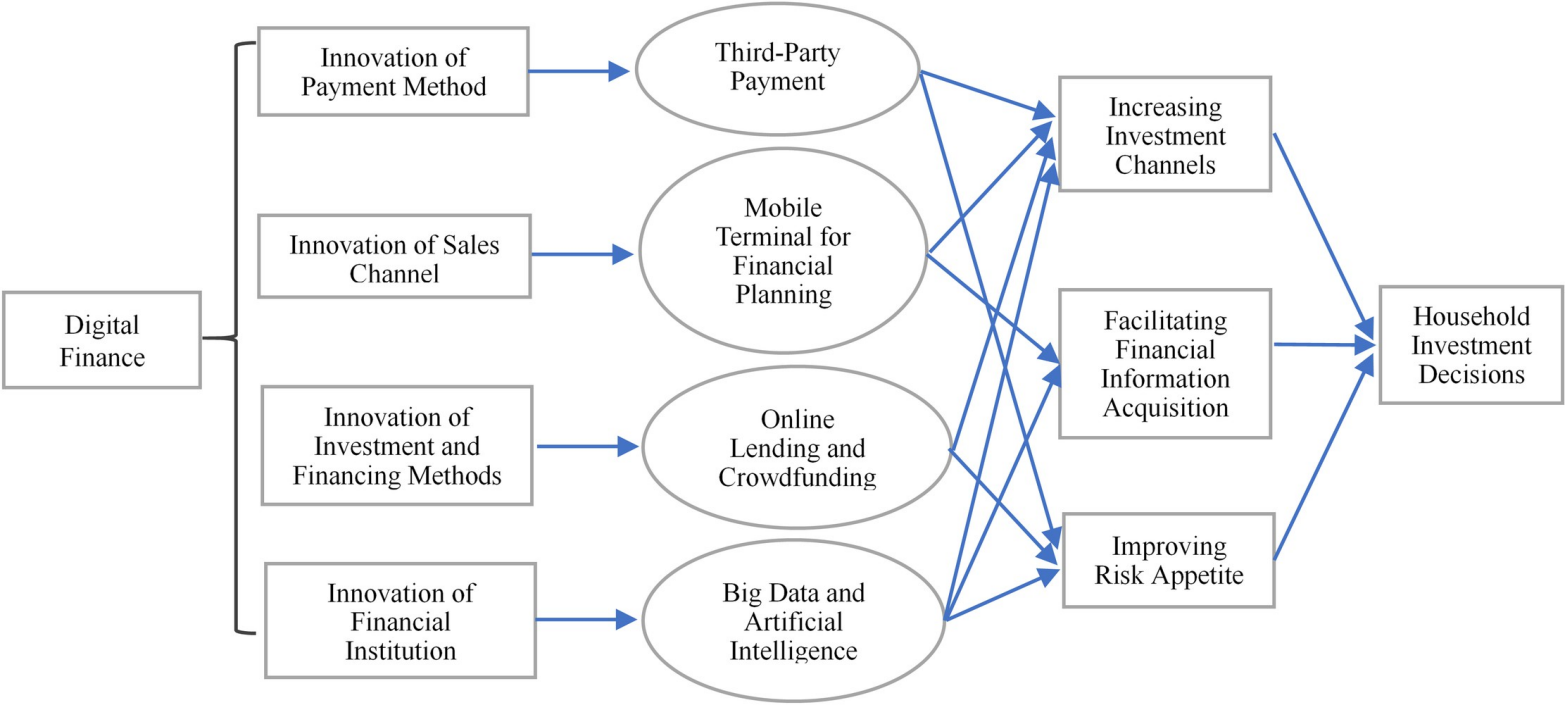
The path analysis of digital finance affecting household risky financial market participation.

According to previous studies, we infer that digital finance mainly promotes household participation in risky financial markets by reducing the lack of investment channels, facilitating financial information acquisition, and raising household risk-bearing levels. In summary, the path of digital finance affecting household risky financial market participation is shown in Fig 1.

First, digital finance provides more convenience for household investment by overcoming time and space constraints. With the help of internet, investors can search for all kinds of investment information online and allocate financial assets anywhere. More importantly, digital finance lowers investment barriers and transaction costs [12], thus expanding the boundaries of financial services to a wider range of “long tail markets” [40]. On the one hand, digital finance has increased family financial channels by providing multiple online payment platforms, such as WeChat, Alipay, and mobile banking. On the other hand, digital finance uses big data and other technological advantages to push relevant product information to investors, which improves the matching degree between financial supply and demand. Therefore, the following is hypothesized:

H1: Digital finance helps promote household participation in risky financial markets.
H2: Digital finance promotes household participation in risky financial markets by reducing the lack of investment channels.

Second, digital finance has prompted investors to pay more attention to financial information. On the one hand, while digital finance provides households with financial products and reduces participation costs, it also stimulates households’ investment demand. Therefore, households pay more attention to financial information actively and improve the possibility of participation in risky financial markets. On the other hand, digital finance has rapidly penetrated into household life. It has promoted social interaction and information sharing among households by providing online trading and communication platforms [51]. Social interaction, as an important way for households to obtain information [52], helps households to cross the investment threshold and allocate financial assets rationally. Therefore, the following is hypothesized:

H3: Digital finance promotes household participation in risky financial markets by facilitating access to financial information.

Third, digital finance can help raise household risk-bearing levels. According to the CHFS data, most Chinese households are risk averse. Research shows that the risk aversion of households severely restricts their participation in the financial market and optimizes asset allocation [53]. Although risk attitude is considered a personal attribute, it changes over time [54]. Currently, digital finance has penetrated every aspect of household life, and digital functions such as mobile payment and online shopping are frequently used. Especially when households get used to the efficient services and rich products of digital finance, they may reduce their risk aversion and increase their risk-bearing level [14], thus promoting households to participate more in risky financial markets. Therefore, the following is hypothesized:

H4: Digital finance promotes household participation in risky financial markets by enhancing their risk-taking level.

## 3 Research design

### 3.1 Data sources

Data used for this study are derived from the 2017 CHFS, a national survey on household finances. The data have been revised biannually since 2011 by the Survey and Research Center for China Household Finance of Southwestern University of Finance and Economics. To ensure the sample’s representativeness, the survey adopts a sampling design combining stratification, the three-stage proportional to scale (PPS) method, and key sampling. The data collection scope contains 25 provinces, municipalities and autonomous regions, 80 counties and 320 communities. The survey collected 8438 households in 2011; the number increased to 28,143 households in 2013. Survey collection continued to expand to 37,289 households in 2015, and reached 40,011 households in 2017. The data collected on household information included demographic characteristics, assets and liabilities, income and consumption, insurance and security, labor, and employment. In terms of household financial assets, the questionnaire covers cash and demand deposits held by the household, whether it owns financial assets such as time deposits, stocks, funds, financial management products, internet financial products, bonds, derivatives, non-RMB assets, and precious metals. Referring to the measurement of household financial participation by previous scholars [55], risky assets mainly include stocks, funds, financial management products, financial bonds, corporate bonds, financial derivatives, non-RMB assets, and precious metal products. This information provides valuable data support to study the issue of household financial market participation. After removing outliers and samples with missing data, we obtain a sample of 10,219 households.

The PKU-DFIIC data are derived from the “Digital inclusive Financial Index of Peking University”, which reflects the development of digital finance [56]. Digital finance refers to the compilation method of the traditional financial inclusion index and combines multiple indicators of digital financial inclusion into a digital financial inclusion index. The aggregate index constructed the index system from three dimensions: the coverage breadth, the use depth and the degree of digitization. These three also constitute the first-level indicators of PKU-DFIIC. First, the coverage breadth embodies one second-level indicator, that is, account coverage. Second, use depth embodies six second-level indicators: payment business, monetary fund business, loan business, insurance business, investment business and credit business. Third, the degree of digitization embodies four second-level indicators: mobilization, affordability, credibility and facilitation. All these second-level indicators also include third-level indicators.

### 3.2 Variables and models

#### 3.2.1 Dependent variables

Household participation in a risky financial market is primarily reflected in two aspects—the breadth and depth of participation. In this study, we take both as dependent variables. The breadth of household participation is measured as the means of participation in a risky financial market, denoted as *risk_if*. Based on the distribution of household participation, this paper sets *risk_if* as a dummy variable. If the households held any risky assets, *risk_if* is equal to 1; otherwise, it is equal to 0. Therefore, the regression analysis is conducted using the probit model as Model (1). *i* denotes households, *digital* denotes the key dependent variable PKU-DFIIC, *X* denotes the set of control variables, and ε denotes a random disturbance term.

$$risk\_if_{i}=1(\beta_{0}+\beta_{1}digital_{i}+\beta_{2}X_{i}+\varepsilon_{i}>0)$$ (1)

The depth of household participation is expressed by the ratio of the number of risky asset classes to the number of financial asset classes held by households, reflecting the concentration of risky asset classes, denoted as *risk_ratio*. Because *risk_ratio* is a left-tailed variable, we discuss the impact of digital finance on the ratio of household risky asset classes held by conducting the tobit model as Model (2).


$$risk\_rate_{i}=\lambda_{0}+\lambda_{1}digital_{i}+\lambda_{2}X_{i}+u_{i}$$ (2)

#### 3.2.2 Core explanatory variable

PKU-DFIIC. This study directly measures the development of digital finance using the “Digital Inclusive Finance Index of Peking University” (*digital*). To further explore which dimension of digital finance impacts household participation, this study also selected the three first-level indicators of the PKU-DFIIC. In addition, to avoid the reverse causality caused by the data of the same period, this study treats all relevant digital financial indices with a two-year lag. Therefore, the endogeneity problems are reduced to some extent. Notably, all relevant digital finance indices are divided by 100 in this study.

#### 3.2.3 Control variables

The current literature lists multiple factors affecting household financial participation [11], we divide the control variables into three categories. First, householder characteristic variables, represented by age, education, gender, marriage, health, insurance, risk preference and risk aversion. Second, household characteristic variables, represented by family size, asset, income and self-employment. Third, regional characteristic variables are represented by rural, Per_bank, Per_GDP and GDP_rate. The specific definitions of the variables are given in Table 1.

**Table 1:** Variable definition and descriptive statistics.

Variables	Definition	Obs	Mean	Std.	Min	Max
Risk_if	Whether to participate in risky financial markets	10,219	0.264	0.456	0	1
Risk_ratio	The number of risky asset classes/the number of total financial asset classes	10,219	0.122	0.204	0	1
PKU-DFIIC	Digital Inclusive Finance Index of Peking University/100	10,219	2.281	0.235	1.933	2.781
Coverage breadth	Breadth of digital finance coverage/100	10,219	2.017	0.287	1.596	2.684
Use depth	Depth of digital finance use/100	10,219	1.855	0.346	1.253	2.598
Digitization level	Digitization level/100	10,219	3.927	0.184	3.738	4.537
Age	The square of age/100	10,219	22.045	9.623	3.240	64
Education	Years of education	10,219	11.619	3.777	0	22
Gender	Householder gender, male: 1, female: 0	10,219	0.84	0.366	0	1
Marriage	Householder marital status, married: 1, others: 0	10,219	0.898	0.303	0	1
Health	Health condition, healthy: 1, unhealthy: 0	10,219	0.928	0.259	0	1
Insurance	Endowment insurance, insured: 1, others: 0	10,219	0.840	0.367	0	1
Risk preference	Householder prefers risk: 1, otherwise: 0	10,219	0.137	0.344	0	1
Risk aversion	Householder avoids risk: 1, otherwise: 0	10,219	0.260	0.439	0	1
Family size	Total household size	10,219	3.373	1.451	0	10
Assets	Ln (household assets)	10,219	13.399	1.248	8.382	16.118
Income	Ln (household income)	10,219	11.251	0.921	6.908	13.453
Self-employment	Self-employed: 1, others: 0	10,219	0.082	0.275	0	1
Household location	Rural: 1, others: 0	10,219	0.182	0.386	0	1
Per_bank	Number of banking financial institutions per 100 km^2^	10,219	10.675	13.946	0.155	64.653
Per_GDP	Ln (per capital GDP)	10,219	11.069	0.39	10.258	11.768
GDP_rate	GDP rate of increase	10,219	0.071	0.014	0.036	0.102

### 3.3 Descriptive statistics

**Fig 2:**
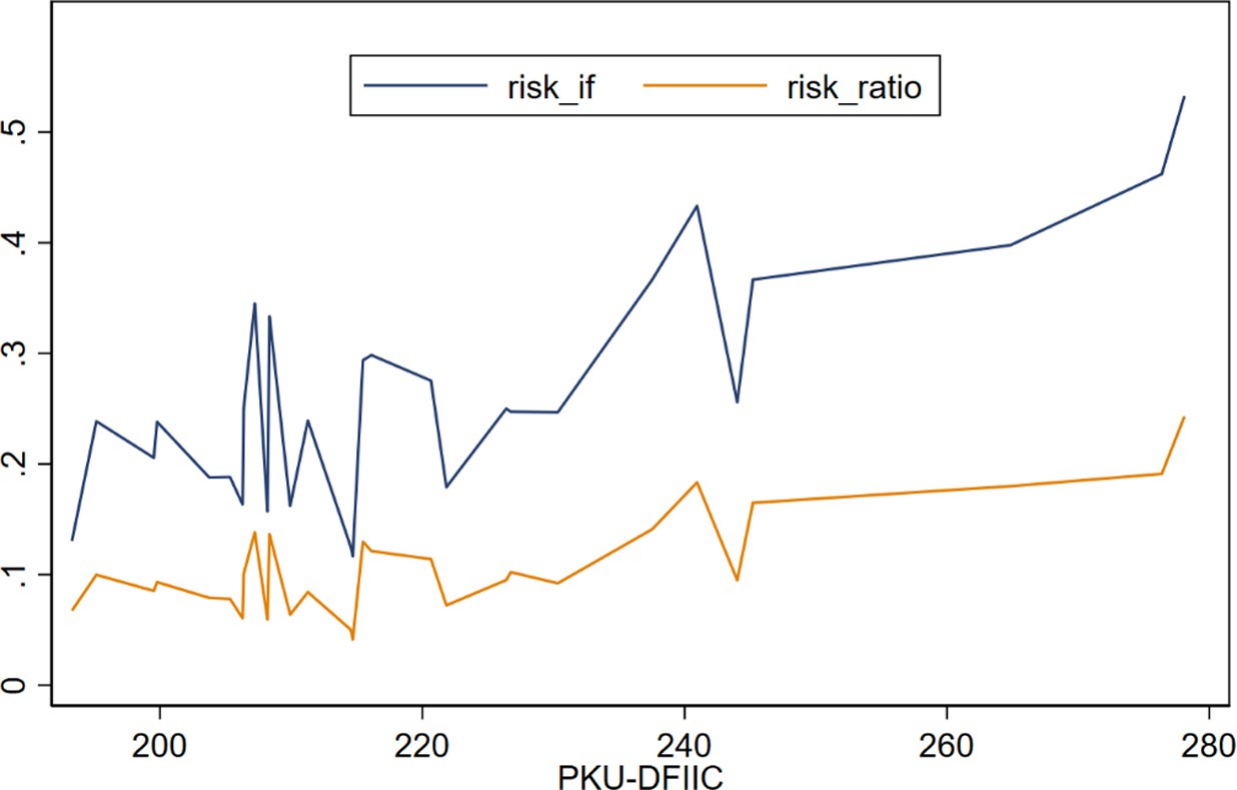
Distribution of digital finance and household participation.

Before proceeding with the analysis, this paper briefly describes household participation in China. Fig 2 illustrates how the household participation rate (*risk_if*) and the ratio of risky asset classes (*risk_ratio*) vary with digital finance. It can be seen that both the participation rate (*risk_if*) and the ratio of risky asset classes (*risk_ratio*) increase with digital finance.

Table 1 shows the descriptive statistics of the main variables. On average, the participation rate of Chinese households in risky financial markets (*risk_if*) in 2017 was 26.4%, and the ratio of risky asset classes held (*risk_ratio*) accounted for 12.2%. These figures suggest that most Chinese households prefer to hold risk-free financial assets, rather than risky assets. The mean value of the core explanatory variable *PKU-DFIIC* is 2.281, with a standard deviation of 0.235. Moreover, the remainder of Table 1 presents the descriptive statistics of the control variables.

## 4 Empirical study

### 4.1 Baseline analysis

#### 4.1.1 The impact of digital finance on household participation in risky financial markets

Table 2 demonstrates the empirical results on the impact of digital finance on household participation in risky financial markets. Columns (1) and (3) report the probit and Tobit regression estimations, respectively. The coefficients are significantly positive at the 1% and 5% levels after controlling for other variables. A 1% increase in digital financial development index (*PKU-DFIIC*) promotes the average probability of household participation in risky financial markets (*risk_if*) by 16.08% and the ratio of risky asset classes held (*risk_rate*) by 8.08%. These results indicate that the development of digital finance makes it significantly more likely for household participation. The impact of other control variables such as age, education and household income are also consistent with the findings of the existing literature [57–59]. For brevity, these coefficients are not reported here.

**Table 2:** Benchmark regressions of PKU-DFIIC on household participation in risky financial market and IV estimation.

Variables	*risk_if*		*risk_rate*	
	Probit	IV-Probit (second stage)	Tobit	IV-Tobit (second stage)
	(1)	(2)	(3)	(4)
PKU-DFIIC	0.1608***	0.4534***	0.0808***	0.1608**
	(0.0385)	(0.1413)	(0.0178)	(0.0686)
Controls	Yes	Yes	Yes	Yes
Obs	10,219	9573	10,219	9573
Pseudo R^2^	0.2579		0.2758	
**First stage**				
IV		-2.9950***		-1.6085**
		(1.1208)		(0.6863)
F value		3291.84		3127.32
Adj R^2^		0.8539		0.8546

Notes: *P < 0.1,

**P < 0.05,

***P < 0.01. Robust standard errors are in parentheses. The estimates of regressions report the average marginal effect.

#### 4.1.2 Endogenous problems

As noted above, although PKU-DFIIC with a lag of two periods is used to reduce the interference of reverse causality, there may still be some endogenous problems. In reference to existing studies [60], we use the spherical distance between the household region and Hangzhou (Digital Finance Center of China) as an instrumental variable. Effective instrumental variables need to meet two conditions: correlation and exogeneity. On the one hand, the farther the household region is from Hangzhou, the lower the development level of digital finance in the region is likely to be. Therefore, it meets the correlation condition. On the other hand, the spherical distance variable constructed from geographical factors does not directly affect household investment behavior. Therefore, it meets exogeneity condition.

Columns (2) and (4) of Table 2 show the results of the second-stage regression using instrumental variable estimation. The coefficients of the PKU-DFIIC remain significantly positive even after accounting for the problem of endogeneity. The test results show that the F statistics of the first-stage IV estimation are all large enough and the instrumental variable is negative and statistically significant at the 1% and 5% levels, indicating that there is no weak instrumental variable selected in this study.

#### 4.1.3 The impact of the first-level indicators of digital finance on household participation in risky financial markets

As digital finance is a multidimensional concept, neither the deepening of internet financial services nor the increase in trading accounts reflects the progress of digital finance [56]. Thus, we select the first-level indicators of PKU-DFIIC including coverage breath, use depth and digitization level to further explore the impact. Table 3 represents the regression results of the first-level indicators of PKU-DFIIC. The coefficients of coverage breath, use depth and digitization level are all significantly positive at the 1% level. The results reveal that multiangle and diversified financial services could better fulfill the needs of households to participate in risky financial markets. In addition, the degree of digitization level has a slightly greater impact, indicating that mobile terminal technologies such as payment have a deeper penetration into people’s lives.

**Table 3:** The regression of the first-level indicators on household participation in risky financial markets.

Variables	*risk_if*	*risk_rate*	*risk_if*	*risk_rate*	*risk_if*	*risk_rate*
	Probit	Tobit	Probit	Tobit	Probit	Tobit
	(1)	(2)	(3)	(4)	(5)	(6)
Coverage breath	0.1123***	0.0541***				
	(0.0309)	(0.0143)				
Use depth			0.1237***	0.0613***		
			(0.0228)	(0.0104)		
Digitization level					0.1532***	0.0695***
					(0.0306)	(0.0145)
Controls	Yes	Yes	Yes	Yes	Yes	Yes
Obs	10,219	10,219	10,219	10,219	10,219	10,219
Pseudo R^2^	0.2576	0.2753	0.2590	0.2769	0.2585	0.2760

Notes: *P < 0.1,

**P < 0.05,

***P < 0.01. Robust standard errors are in parentheses. The estimates of regressions report the average marginal effect.

### 4.2 Heterogeneity analysis

Due to the heterogeneity of households, there may be certain group and regional differences in the impact of digital finance on household investment behavior. Therefore, this section presents the heterogeneity in terms of household wealth, education level, and household location.

Table 4 reports the impact of digital finance on household participation by wealth size. Referring to the practice of previous literature [61], we divide the sample households into low-wealth group, medium-wealth group and high-wealth group according to the household asset size. The regression results reveal that digital finance has significant positive impact on households with different levels of wealth. The results suggest that the development of digital finance effectively reduces the deterrent effect of wealth thresholds on household participation in risky financial markets. Thus, on the one hand, households with less wealth obtain more investment opportunities because digital finance has lowered the investment entry threshold. On the other hand, households with more wealth can give full play to their asset advantages with the help of digital finance.

**Table 4:** Heterogeneity analysis 1: Household wealth.

Variables	Low-wealth		Medium-wealth		High-wealth	
	Probit	Tobit	Probit	Tobit	Probit	Tobit
	(1)	(2)	(3)	(4)	(5)	(6)
*PKU-DFIIC*	0.1165**	0.0571***	0.3273***	0.1624***	0.2109*	0.1389*
	(0.0456)	(0.0193)	(0.0826)	(0.0398)	(0.1273)	(0.0781)
Controls	Yes	Yes	Yes	Yes	Yes	Yes
Obs	6340	6340	2644	2644	1235	1235
Pseudo R^2^	0.1984	0.2079	0.1543	0.1726	0.1475	0.1773

Notes: *P < 0.1,

**P < 0.05,

***P < 0.01. Robust standard errors are in parentheses. The estimates of regressions report the average marginal effect.

Table 5 presents the impact of digital finance on householders with different levels of education. According to the instructions of the Chinese Ministry of Education, we divide the sample householders into a low-education group, a medium-education group and a high-education group. The regression results highlight the positive impact of digital finance on householders at all levels of education. This suggests that the development of digital finance effectively reduces the deterrent effect of cognitive thresholds on household participation in risky financial markets. Thus, on the one hand, householders with lower education can overcome the barrier to asset allocation raised by ignorance and misperceptions because digital finance has lowered the cognitive threshold. On the other hand, digital finance enables households that are not limited by cognitive thresholds to better leverage their strengths in risky financial market.

**Table 5:** Heterogeneity analysis 2: Education level.

Variables	Low-education		Medium-education		High-education	
	Probit	Tobit	Probit	Tobit	Probit	Tobit
	(1)	(2)	(3)	(4)	(5)	(6)
PKU-DFIIC	0.0981**	0.0456***	0.1518*	0.0729*	0.2454***	0.1338***
	(0.0445)	(0.0173)	(0.0855)	(0.0384)	(0.0812)	(0.0420)
Controls	Yes	Yes	Yes	Yes	Yes	Yes
Obs	4378	4378	2394	2394	3447	3447
Pseudo R^2^	0.1649	0.1676	0.1584	0.1710	0.1230	0.1387

Notes: *P < 0.1,

**P < 0.05,

***P < 0.01. Robust standard errors are in parentheses. The estimates of regressions report the average marginal effect.

Table 6 displays the impact of digital finance on household location. The regression results reveal that digital finance played a positive role in both rural and urban areas in promoting household participation in risky financial markets. In addition, the effect of digital finance on urban households is slightly larger than that on rural households. We infer that infrastructure, such as the internet, is better established in urban areas and is coupled with better education and higher wealth. Thus, financial supply plays a significant role in promoting digital finance [60].

**Table 6:** Heterogeneity analysis 3: Household location.

Variables	Rural		Urban	
	Probit	Tobit	Probit	Tobit
	(1)	(2)	(3)	(4)
PKU-DFIIC	0.1566***	0.0658***	0.1609***	0.0798***
	(0.0456)	(0.0223)	(0.0539)	(0.0214)
Controls	Yes	Yes	Yes	Yes
Obs	1857	1857	8362	8362
Pseudo R^2^	0.1871	0.1907	0.2117	0.2281

Notes: *P < 0.1,

**P < 0.05,

***P < 0.01. Robust standard errors are in parentheses. The estimates of regressions report the average marginal effect.

### 4.3 Mechanism analysis

This section examines possible mechanisms by which digital finance affects household participation in risky financial markets. We evaluate the influences of digital finance from three perspectives: whether they feel they lack access to investment channels, whether they pay attention to economic information and whether they prefer risks. These questions are asked in the 2017 CHFS. Based on the responses, we define the mediating variables. First, we define the variable *lack of investment channels*. The variable takes a value of 1 if the household perceives a lack of investment channels, and a value of 0 otherwise. Columns (1) and (2) of Table 7 show that the development of digital finance significantly reduces the possibility of lack of household investment channels. The results indicate that the development of digital finance has broken through the dependence of household investment on traditional physical financial outlets, and has increased the convenience of investors in time and space [62]. Second, we define the variable *attention to economic information*. The variable takes a value of 1 if the household pays attention to economic information, and a value of 0 otherwise. Columns (3) and (4) of Table 7 show that the development of digital finance significantly promotes households’ access to financial information, which in turn promotes households’ participation in risky financial asset allocation. Third, we define the variable *risk prefer*. The variable takes a value of 1 if the household is risk preference, and a value of 0 otherwise. Columns (5) and (6) of Table 7 show that the development of digital finance significantly increases the possibility of household risk appetite and thus households more actively participate in risky financial markets. By combining the results, we show that developing digital finance helps households believe that there are plentiful investment channels and economic information, and may incentivize them to improve their risk appetite in an attempt to participate in risky financial markets.

**Table 7:** Mechanism analysis.

Variables	Lack of investment channels		Attention to economic information		Risk prefer	
	Probit	Tobit	Probit	Tobit	Probit	Tobit
	(1)	(2)	(3)	(4)	(5)	(6)
PKU-DFIIC	-0.0034*	-0.0035*	0.0170**	0.0175**	0.0850**	0.0876**
	(0.0019)	(0.0021)	(0.0073)	(0.0077)	(0.0342)	(0.0356)
Controls	Yes	Yes	Yes	Yes	Yes	Yes
Obs	7739	7739	10,219	10,219	10,219	10,219
Pseudo R^2^	0.0228	0.0163	0.0819	0.0630	0.0448	0.0345

Notes: *P < 0.1,

**P < 0.05,

***P < 0.01. Robust standard errors are in parentheses. The estimates of regressions report the average marginal effect.

### 4.4 Robustness checks

To examine the robustness of the benchmarking analysis, we substitute the original regression sample from two perspectives. First, we re-estimate the impact of digital finance, after excluding households engaged in the financial industry from the sample. Considering that these households have more access to financial markets, they are more likely actively to participate in investments [63]. Therefore, we excluded these samples and reported the regression results. Columns (1) and (2) of Table 8 show that the coefficients of PKU-DFIIC are significantly positive at the 1% level, suggesting that digital finance still promotes household participation in risky financial markets, consistent with the results in Table 2. Second, we re-estimate the impact of digital finance using the 2015 CHFS survey sample. Columns (3) and (4) of Table 8 show that the coefficients of PKU-DFIIC are significantly positive at the 1% level, suggesting that digital finance still promotes household participation in risky financial markets, consistent with the results in Table 2.

**Table 8:** Robustness checks.

Variables	Robustness Check 1: Exclude households engaged in the financial industry		Robustness Check 2: Replaced with the 2015 survey data	
	*risk_if*	*risk_rate*	*risk_if*	*risk_rate*
	Probit	Tobit	Probit	Tobit
	(1)	(2)	(3)	(4)
PKU-DFIIC	0.1634***	0.0820***	0.1628***	0.0890***
	(0.0398)	(0.0181)	(0.0175)	(0.0106)
Controls	Yes	Yes	Yes	Yes
Obs	9490	9490	12,677	12,677
Pseudo R^2^	0.2512	0.2692	0.2855	0.2643

Notes: *P < 0.1,

**P < 0.05,

***P < 0.01. Robust standard errors are in parentheses. The estimates of regressions report the average marginal effect.

## 5. Conclusions

Based on the data from the CHFS and the PKU-DFIIC, this study examined whether digital finance exerts an impact on household participation in risky financial markets. The findings of this study are as follows. First, digital finance significantly promotes household participation in risky financial markets. This outcome holds after accounting for the endogeneity problems. Second, digital finance affects household participation through three channels: reducing the lack of investment channels, promoting households’ access to financial information and increasing the possibility of household risk appetite. Third, the development of digital finance effectively reduces the deterrent effect of wealth and cognitive thresholds on household participation in risky financial markets. This likely reflects the nature of inclusive finance because it provides households with low wealth and cognition to access the financial market, while it enables households that are not limited by wealth and cognitive thresholds to better leverage their strengths.

This study also has significant practical implications according to the findings. We propose the following policies for government financial reforms to further develop financial market. First, we offer a way to promote household participation in risky financial market by extending the development of digital finance, especially in relation to the digitization level. Second, the impact of imbalanced developed digital finance on different groups still exists, even though they are all beneficiaries of digital finance. The heterogeneity analysis shows both the inclusive function of digital finance and the digital divide. Households with low wealth and education, as well as those living in rural areas need more attention [62], which offers the government a direction for a coordinated strategy with financial institutions, to promote disadvantaged households’ financial education and innovate more suitable financial products. Third, internet financial management has played an important role in promoting household participation in risky financial markets due to its diversity and convenience. Relevant departments should promote the healthy development of such markets, further prosper the financial supply market, and improve the level of financial services. Finally, efforts must be made to strengthen the safety of digital finance. As digital finance has penetrated all levels of society, raising the possibility of risk transmission [64], policymakers need to step up efforts to improve regulation of digital finance and control risks to eliminate online fraud and protect households’ legal interests.

Although we tried our best to investigate the possible channels by which digital finance influences household investment behavior, we recognize other factors and influencing channels that we did not consider may still exist. In addition, the development of digital finance has not gone through a complete cycle, and many problems have not been fully exposed. Further research should attempt to explore more influence channels. At the same time, we need to pay close attention to the development of digital finance at any time and further study household investment behavior in risk management, investment return and other aspects.

## Supporting information

S1 Table (XLSX)
